# Altered Promoter and G-Box Binding Factor for *1-Deoxy-d-Xylulose-5-Phosphate Synthase* Gene Grown from *Poa pratensis* Seeds after Spaceflight

**DOI:** 10.3390/ijms20061398

**Published:** 2019-03-21

**Authors:** Lu Gan, Yuehui Chao, Haotian Su, Yujing Ren, Shuxia Yin, Liebao Han

**Affiliations:** Institute of Turfgrass Science, Beijing Forestry University, Beijing 100083, China; ganlu2016@bjfu.edu.cn (L.G.); chaoyuehui@163.com (Y.C.); suhaotian@bjfu.edu.cn (H.S.); ren950922@163.com (Y.R.)

**Keywords:** deletion, mutation after spaceflight, promoter region, *1-deoxy-d-xylulose-5-phosphate synthase 1*, G-box binding factor, *Poa pratensis*

## Abstract

In plant cells, the nucleus DNA is considered the primary site of injury by the space environment, which could generate genetic alteration. As the part of genomic mutation, genetic variation in the promoter region could regulate gene expression. In the study, it is observed that there is a deletion in the upstream regulatory region of the *1-deoxy-d-xylulose-5-phosphate synthase 1* gene (*PpDXS1*) of *Poa pratensis* dwarf mutant and the *PpDXS1* transcript abundance is lower in the dwarf mutant. It is indicated that the deletion in the promoter region between wild type and dwarf mutant could be responsible for the regulation of *PpDXS1* gene expression. The *PpDXS1* promoter of dwarf mutant shows a lower activity as determined by dual luciferase assay in *Poa pratensis* protoplast, as well as the *GUS* activity is lower in transgenic *Poa pratensis* plant. To further investigate the effect of the deletion in the promoter region on *PpDXS1* transcript accumulation, the transient assay and yeast one-hybrid experiment demonstrate that the deletion comprises a motif which is a target of G-box binding factor (GBF1), and the motif correlates with an increase in transactivation by GBF1 protein. Taken together, these results indicate that the deletion in the promoter of *PpDXS1* isolated from dwarf mutant is sufficient to account for the decrease in *PpDXS1* transcript level and GBF1 can regulate the *PpDXS1* gene expression, and subsequently affect accumulation of various isoprenoids throughout the plant.

## 1. Introduction

Terpenoids are a large group of primary and secondary metabolites involved in plant life processes, and there is mounting evidence that metabolites such as phytohormones and chlorophyll can regulate their growth and development [1,2]. Historically, some monoterpene, diterpene, and other secondary metabolites are derived from plastidic methyl erythritol phosphate (MEP) pathways [3]. In the literature, 1-deoxy-d-xylulose-5-phosphate (DXP) synthase (DXS) can broadly defined as the first limited enzyme of the MEP pathway [4]. The *DXS* gene was first introduced in *Escherichia coli* and *DXS* homologs were subsequently found in model plants and crops [5,6]. Previous studies showed that DXS is encoded by a small gene family and two or three *DXS* homologs generally divided into three independent clades [7,8]. The *DXS1* gene is proved to be involved in primary metabolism (e.g., chlorophyll synthesis, photosynthetic processes), and mainly expressed in photosynthetic tissues [7,9]. On the other hand, the *DXS2* and *DXS3* genes in various plants are thought to be involved in biotic or abiotic resistance defenses and produce special secondary metabolites in defense responses [6,10].

Space mutation factor, including the microgravity, cosmic radiation, hypomagnetic field and vibration, etc., can trigger non-directional variation in plants. Cosmic radiation, as one of the main factors can produce genetic changes in seeds and meristems as a result of phenotypical alterations and metabolism [11]. In plant cells, the nucleus DNA is considered the principal site of injury by radiation, which is responsible for non-targeted damage and generates different kinds of mutations, such as base deletions, base substitutions, translocations, inversions and large insertions [12,13]. Due to the high conservation of most functional genes, they are less prone to mutagenesis or easily repaired after mutation by the space environment. But mutations in the upstream promoter of genes will affect gene expression, which lead to changes in the gene function associated with plant growth and development. It is known that gene expression is controlled at many levels with key regulatory elements being located in non-coding regions of the genome, such as promoter, intron, and 3′ UTRs [14]. Besides, gene expression-associated variants within regulatory regions are anticipated to mediate an effect on trans-acting regulatory factors (e.g., transcription factors and miRNAs). For example, the insertion of transposable elements in the promoter region of maize genes exhibit activation of gene expression [15]. Another example by Mernke et al. [16] was that the rearrangement caused by insertion of a retroelement-derived sequence in the promoter region of *major facilitator superfamily transporter* gene (*mfsM2*) resulted in its overexpression, which is responsible for the appearance and subsequent spread of multidrug resistance in vineyards. Besides, Espley et al. [17] also reported that a rearrangement in the upstream regulatory region of the *myeloblastosis 10* (*MYB10*) gene in apple is responsible for the increase in MYB10 transcript levels and subsequent ectopic accumulation of anthocyanins.

There are many examples of post-transcriptional and post-translational regulation (e.g., external factors) of *DXS* gene expression and enzyme activity [5,18]. Therein, a number of cis-acting elements in *DXS* promoter play an important role in the transcriptional regulation of *DXS* gene expression in various plants, such as sequences over-represented in light-induced promoters (SORLIP1-AT) associated with light-regulation, GATA box (a gibberellin-responsive element) and etc. [19,20]. Although several studies regarding *DXS* gene expression and the cis-regulatory elements of *DXS* promoter have been performed, the relationship between the insertion/deletion in the promoter of *DXS* gene caused by space mutation and its gene expression have not been reported, which is worth further investigation.

Here, we reported a 500 bp-fragment in the upstream regulatory of *DXS1* gene found only in the wild type of Kentucky bluegrass (*Poa pratensis* L., KB), but it is lacking in the dwarf mutant derived from the space-exposed KB seeds. In the previous study, the transcript abundance of *DXS1* gene in the wild type is higher than in the dwarf mutant and there was no difference in the amino acid sequence of *DXS1* isolated from the two plants. However, we would like to know whether the *PpDXS1* gene expression is associated with the difference of the promoter region. By dual luciferase assay and genetic transformation in KB, the *PpDXS1* promoter isolated from the wild type contained the functional 500 bp-deletion showed a higher activity in protoplast, as well as the *GUS* expression was higher in transgenic KB constitutively expressed the promoter from wild type. Also, there is an important motif binding with GBF1 (G-box binding factor) protein in the 500 bp-deletion fragment. By transient assay and yeast one-hybrid experiment, the transcription factor could activate the *PpDXS1* transcript accumulation, leading to the subsequent accumulation of various isoprenoids throughout the plant.

## 2. Result

### 2.1. Isolation of Upstream Regulatory Region of PpDXS1

**Figure 1:**
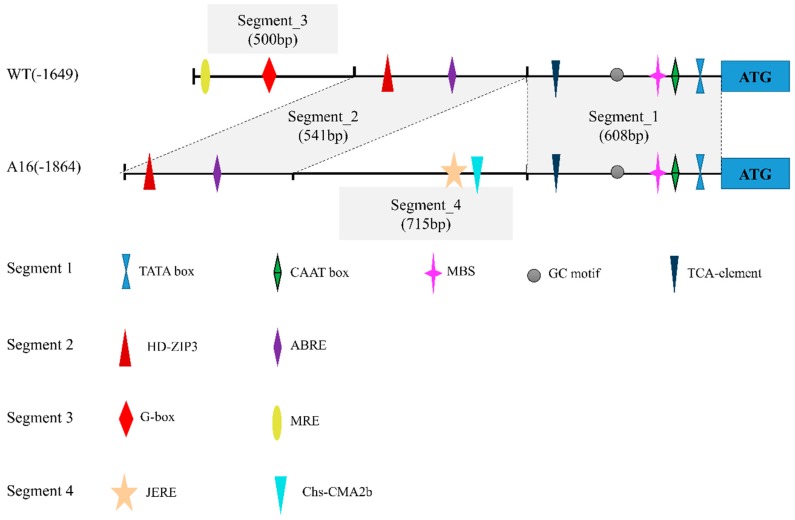
*PpDXS1* promoter from *P. pratensis* wild-type (WT) and dwarf mutant (A16). Putative cis elements are labeled as shown. Element positions are shown proportional to the full length of the promoter. The common segment of promoter regions in WT and A16 are shaded.

To investigate the molecular basis of the phenotype of dwarf mutant induced by spaceflight, genome walking was applied to isolated the upstream sequence of *PpDXS1*. Sequences of upstream region were cloned from both KB’s wild type (WT) and dwarf mutant (A16), whose lengths were 1649 bp and 1864 bp, respectively (Figure 1). The isolated DNA fragments revealed two sequences of different component and rearrangement in the full length, while the two different sequences share two same segments as shown in Figure 1. The segment 3 (500 bp) was present in the wild type (WT) but absent in dwarf mutant (A16). Besides, the other fragment contained an insertion of 715 bp (segment 4).

A motif search of the two promoter sequences was performed using the PlantCARE databases to reveal putative cis element. Except the core promoter element (TATA-box, CAAT-box, and GC-motif), other elements that corresponded to light response, hormone metabolism, and stress defense regulatory were found (Figure 1). They are: MBS (MYB binding site involved in drought-inducibility), ABRE element (abscisic acid response factor), JERE (related to jasmonic acid and ethylene responsiveness), TCA element (involved in salicylic acid responsiveness), MRE and Chs-CMA2b (involved in light responsiveness), and protein binding sites (G-box, HD-ZIP3). As shown in Figure 1, sequences in promoter segment 1 and 2 were similar by over 95% and had common elements, while the segment 3 comprised several unique cis elements (e.g., G-box) in WT that were not found in A16 (Table S1). Analysis of these different segments and specific motifs of the *PpDXS1* promoter region may identify the functional significance of regulatory elements for the expression of the *PpDXS1* gene and thus provide some clues for the dwarf mechanism by this gene.

### 2.2. Activity of PpDXS1 Promoter in the Dual Luciferase Transient Protoplast Assay

**Figure 2:**
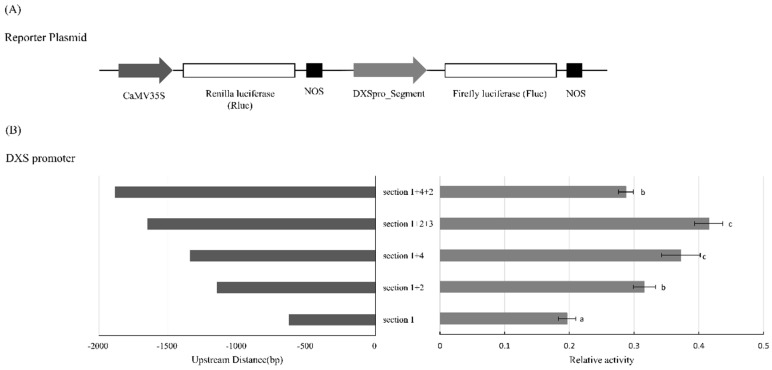
Transformation of *Poa pratensis* protoplasts with different segment of *DXS1* promoter region fused to firefly luciferase (*Fluc*). (**A**) Schematic representation of DXSpro_segment: Fluc vector construction. CaMV 35S: Renilla luciferase (*Rluc*) served to the normalized control, NOS is the terminator; (**B**) the left presents the length of different segment or combination in the *DXS1* promoter region. The right is the presence of relative Fluc activity (normalized by Rluc activity), as the segment_1 set to control. Data represent the average value with standard error of three biological replicates. Different letters above the bars indicate significant difference (*p* < 0.05).

One possible result of the different segments or rearrangement within the promoter of *PpDXS1* in WT and A16 is an alteration in the basal activity of the promoter. A preliminary experiment has shown that when the two versions of full-length promoter were fused to the enhanced green fluorescent protein (*EGFP*) into KB protoplast, the green fluorescent could be detected under the fluorescence microscope. Although the activities of the two promoters were both observed, whether there are differences within full or part of promoter in activity requires quantitative verification. A dual luciferase assay was used to quantify the activity of the different segment or group of the *PpDXS1* promoter. The full-region and different segment of *DXS1* promoter, including segment_1, segment_1+2, segment_1+4, segment_1+2+3 and segment_1+4+2, were individually fused to firefly luciferase (*Fluc*) and transactivation of the *Fluc* gene measured relative to renilla luciferase (*Rluc*) under the control of the cauliflower mosaic virus 35S promoter (CaMV 35S) by measurement of luminescence after transient expression in KB protoplast (Figure 2A). There are a number of important differences in relative activity of various promoter segments (Figure 2B). The relative activity of DXSpro_WT_*Fluc* tends to have positive correlation with the promoter length, while the DXSpro_A16_*Fluc* varied with segment arrangement in the dwarf mutant. Besides, the relative activity of segment_1+4+2 was lower than the segment_1+2+3, as well as the segment_1+4 (Figure 2B).

### 2.3. Promoter Activity in Poa pratensis Plants

**Figure 3:**
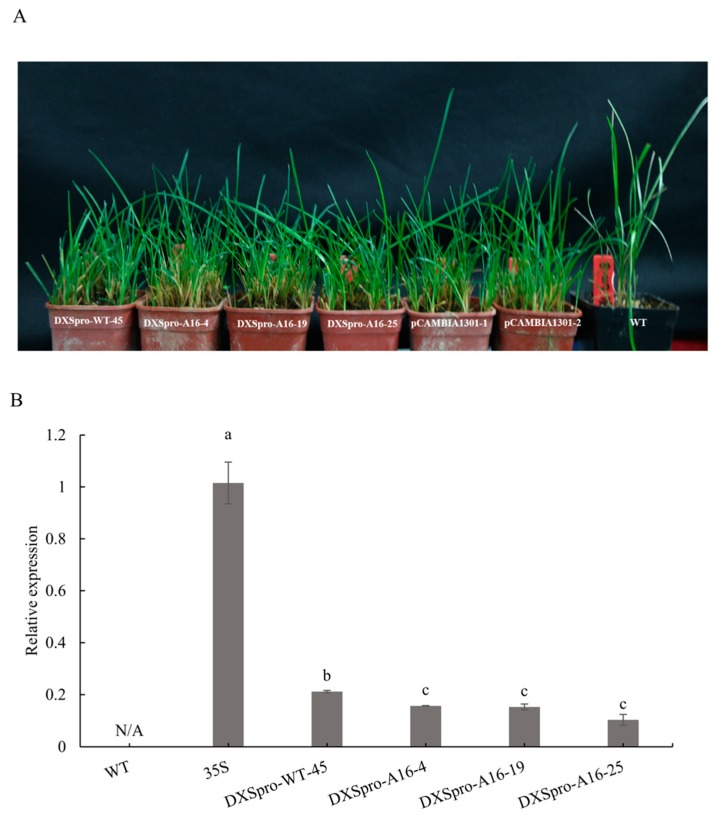
Analysis of *PpDXS1* promoter in transgenic *Poa pratensis* plants. (**A**) Photos of plants with transformed *PpDXS1* promoters from WT and A16, and pCAMBIA1301 vector; (**B**) relative expression of *GUS* gene in transgenic lines of different *PpDXS1* promoters. The *Actin* is reference gene, and the control plant is transformed pCAMBIA1301-1 line. All quantitative reverse transcription polymerase chain reactions (qRT-PCR) were performed from triplicate biological samples. The 2^−ΔΔ*C*t^ method was used to calculate the fold expression relative to the control. Mean of three replicates ± standard error is shown. Different letters above the bars indicate significant difference (*p* < 0.05). N/A, not applicable.

Next, we analyzed the promoter in *Poa pratensis*. The two version of full-length *DXS* promoters were cloned into a binary vector and fused downstream of a *GUS* reporter gene and introduced into *Poa pratensis*. The CaMV 35S promoter fused to the *GUS* gene served as the positive control. A variety of independent transgenic lines were generated through particle bombardment transformation, including one DXSpro_WT line, three DXSpro_A16 lines and two pCAMBIA1301 lines (Figure 3A). *Pgk1*, a single-copy gene in Poaceae family, was used to evaluate the copies of *GUS* gene in transgenic lines by quantitative RT-PCR. These results showed that total transgenic lines of inserted DXSpro is single copy (Table S4).

Firstly, the activity of the promoters was examined through GUS histochemical staining in transgenic plants (Figure S1). The staining results showed that GUS activity is detected in the transgenic lines, but GUS activity of the DXSpro_WT line was higher than that of DXSpro_A16 and both of them were lower than that of CaMV 35S promoter. In order to further evaluate the activity of the *DXS1* promoters, we compared the expression level of the *GUS* gene in the leaves of *DXSpro-WT:GUS*, *DXSpro-A16:GUS* and *CaMV35S:GUS* plants. We observed that the *GUS* gene expression in transformed *DXSpro-A16:GUS* lines was significantly decreased compared with *DXSpro-WT:GUS* (Figure 3B). These results support the fact that the deletion and/or insertion in *PpDXS1* promoter are presented with different transcriptional levels in *Poa pratensis*.

### 2.4. Enhanced Activity by Addition of GBF1 to DXS1 Promoter

**Figure 4:**
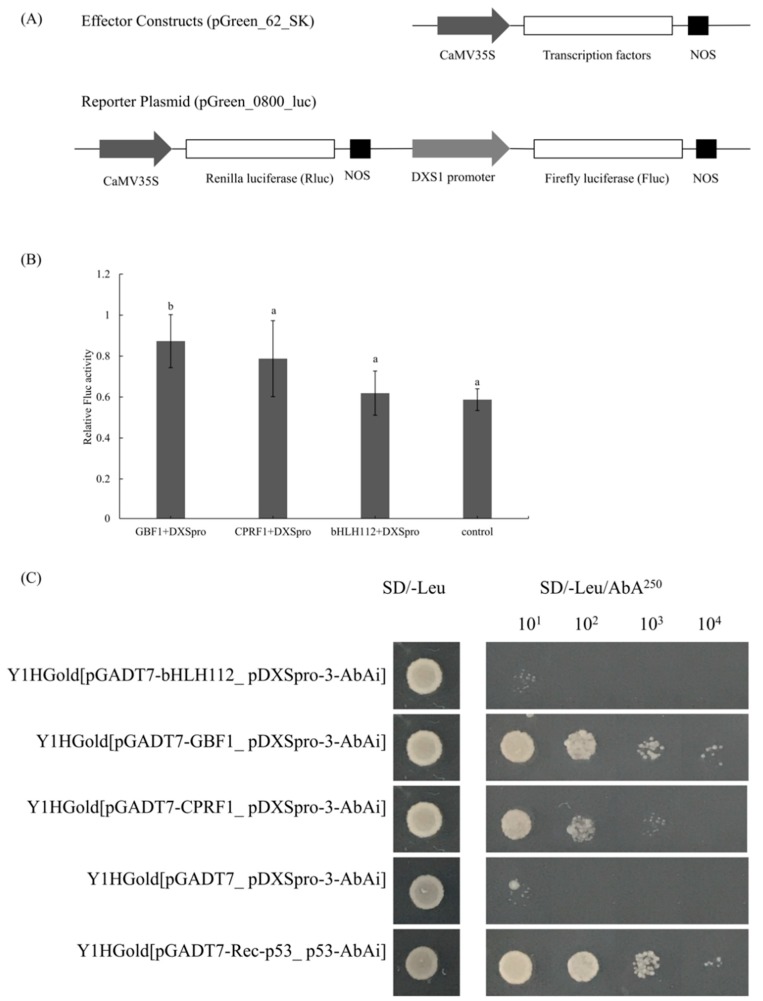
PpGBF1 transcription factor interacts with the deletion of *PpDXS1* promoter region from A16. (**A**) Schematics of the normalization control, reporter, and effector construction. In the reporter constructs, the firefly luciferase (*Fluc*) gene was placed under the control of *DXS1* promoter from WT. CaMV 35S: transcription factors (TFs), including *35S:GBF1*, *35S:CPRF1*, and *35S:bHLH112*, served as three effector constructs, respectively. The empty effector vector was used as a negative control; (**B**) the relative Fluc activity (normalized by Rluc activity) of TFs cooperated with DXSpro_WT_Fluc in the control of the empty effector. Data represent the average value ± standard error of three biological replicates. Different letters above the bars indicate significant difference (*p* < 0.05); (**C**) Interaction between segment_3 of *PpDXS1* promoter and each transcription factor (PpbHLH112, PpGBF1, and PpCPRF1, from top to bottom) was respectively tested based on the ability of transformed Y1H yeast to grow on SD/-Leu/AbA^250^ medium in the gradient dilution. The Y1H_pGADT7_pDXSpro-3-AbAi was used as the positive control, and the Y1H_pGADT7-Rec-p53_p53-AbAi is the negative control.

To identify any possible cis-elements located within the functional region of *PpDXS1* promoter, we surveyed the DXSpro_WT and DXSpro_A16 region for the presence of known transcriptional factor binding sites using the online TRANSFAC and JASPAR database (Table S2). Therein, a G-box element (CACGAT) was only observed at the -1455 positions (segment 3) in the DXSpro_WT region (Figure 1). G-box binding protein is a big family, which are important TFs regulating photosynthesis, including G-box binding factor 1 (GBF1), common plant regulatory factor 1 (CPRF1) etc. Moreover, a binding site of basic helix-loop-helix factor (bHLH112) was also predicted in the segment_1. To determine whether the GBF1 and bHLH112 proteins are involved in the induction of the *DXS* promoter and subsequently regulation of the expression of *PpDXS1*, we examined the additional effect of these transcription factors to *DXS1* promoter activity (Figure 4A). When segment_1+2+3 of *DXS1* promoter isolated from the wild-type plant was used as the reporter, the enhanced activity from the import of PpGBF1 transcriptional factors to DXSpro_WT was about 1.4-fold higher compared with the control, while PpCPRF1 and PpbHLH112 protein have no significant effect on the promoter activity (Figure 4B). This induction of GBF1 to *DXS1* promoter demonstrated that GBF1 acts positively on the transcriptional level, which supports the hypothesis that the segment or elements lacking in A16 were responsible for *DXS1* promoter activity.

To confirm further the DNA binding ability of PpGBF1, PpCPRF1 and PpbHLH112, we performed a yeast one-hybrid (Y1H) assay in which these transcription factors were examined for different segments of the promoter region. Our Y1H assays showed that the PpGBF1 directly interacts with segment_3 of the *DXS1* promoter region (Figure 4B). No interaction was detected between PpCPRF1 and each segment of promoter, although the yeast was grown in the first dilution. Besides, PpCPRF1 and PpbHLH112 did not interact with whether deletion or insert segment of *DXS1* promoter (Figure S2). This is consistent with the result of dual luciferase assay. Taken together, these findings indicate that PpGBF1 binds to the deleted segment of DXS1pro_A16, and presumably the transcription factor elevated the *DXS1* gene expression.

### 2.5. Overexpression of GBF1 Gene in Poa pratensis

**Figure 5:**
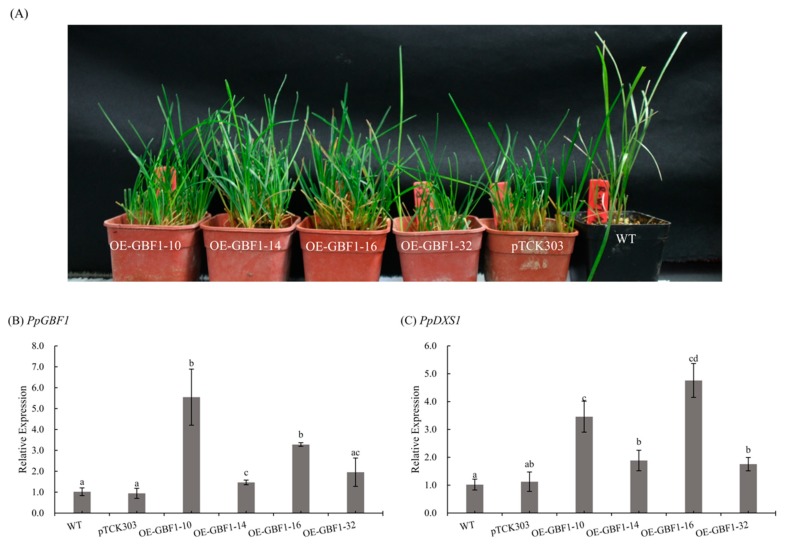
Characterization of *PpGBF1* transgenic plants. (**A**) Pictures of OE-*PpGBF1* transgenic lines. From left to right, these are OE-*GBF1*-10, OE-*GBF1*-14, OE-*GBF1*-16, OE-*GBF1*-32, pTCK303 control, and wild type; (**B**) relative expression of *PpGBF1* gene in transgenic lines; (**C**) relative expression of *PpDXS1* gene in transformed OE-*GBF1* plants. The Actin is the reference gene, and the control plant is transformed pTCK303 empty vector line. All qRT-PCR reactions were performed from triplicate biological samples. The 2^−ΔΔ*C*t^ method was used to calculate the fold expression relative to the control. Mean of three replicates ± standard error is shown. Different letters above the bars indicate significant difference (*p* < 0.05).

To study the function of *GBF1* gene in *Poa pratensis*, we generated transgenic plants overexpressing (OE) *PpGBF1*. Quantitative reverse transcription polymerase chain reaction (qRT-PCR) was used to examine the expression level of *PpGBF1* in OE transgenic plants. As shown in Figure 5B, expression level of *PpGBF1* were significantly increased in all transgenic lines except OE-GBF1-32 line. To determine whether GBF1 contribute to the induction of the *DXS1* gene in *Poa pratensis*, the transcript level of *PpDXS1* gene was analyzed in wild type, control and transgenic plants. As shown in Figure 5C, the transcript of *DXS1* accumulated in the OE-GBF1-10 and OE-GBF1-16 line, demonstrating that GBF1 acts positively on the expression of the *DXS1* gene.

## 3. Discussion

In our previous work, *Poa pratensis* seeds were carried on a space flight for 18 days on the 20th returnable satellite and returned to the ground to plant together with the wild type in the same growth conditions. Besides, we isolated *1-deoxy-d-xylulose-5-phosphate synthase 1* gene (*PpDXS1*) from *Poa pratensis* wild-type (WT) and dwarf mutant (A16) plant respectively and found no significant mutation in the coding region, while the *PpDXS1* gene expression is higher in the WT compared with A16. So, we wonder what else decreased the *PpDXS1* gene expression. In this study, the promoter sequences of *PpDXS1* gene were isolated from two cultivars by hi-Tail-PCR technology. Sequence analysis showed that, compared with WT, 715-bp insertions and 500-bp deletions in the promoter region were identified in A16 (Figure 1). It is speculated that the mutation after spaceflight induced these insertion and deletion of fragments in the *DXS1* gene promoter region. Other studies also showed that space environmental factors (e.g., ionizing radiation and microgravity) can produce induced mutations in the plant genome, such as insertion or deletion [21,22,23,24]. Whether insertion or deletion occur in coding sequences or in non-coding regions (e.g., promoter and gene spacing regions), it is prone to affect gene expression and phenotype [25,26,27]. For example, the 193-bp insert and 397-bp deletion in the promoter region of *Hordeum vulgare waxy* gene altered transcription, therein 397-bp deletion probably resulted in the decreased granule-bound starch synthase [28].

We used a transient assay to analyse the promoter activity of the differential and common segments from two cultivar sequences, to estimate which variation would affect the expression of *DXS* gene by fusing a *Fluc* reporter in *Poa pratensis* protoplast. From partial segments to complete the promoter, the transcriptional activity of the promoter from the WT was increased as the length in the transient assay (Figure 2B), while the relative activity of A16 complete promoter sequence was not only lower than WT, but also the insertion with the common fragments itself (segment_1+4, Figure 2B). It is indicated that these sequences were involved in the deletion and/or insertion and the functional binding of transcription factors, so we would focus on these different segments.

Besides, the construct of native promoter with *GUS* reporter built to study the effect of different promoter in the stable expressed *Poa pratensis*. In the data set presented in Figure 3, all the transgenic lines of A16 promoter showed a reduction in the transcriptional level of the *GUS* gene compared with the WT promoter transformed plants. One explanation is that some suppressed motif was formed at the junction between the insertion and common fragment (segment_1/2). However, after the search of the A16 promoter sequence in the PlantCARE database, we did find the published motifs or binding sites with inhibitor effect. It is necessary to investigate and verify the existence of inhibitory sites in the future. Another explanation may be that deletion in the promoter region of the dwarf mutant contains protein binding sites that enhance transcriptional activity. There have been a considerable number of investigations associating 5′-upstream deletion analysis with transcriptional regulation [29,30]. Atanassova et al. [31] demonstrated that deletion of the upstream 558 bp of *histone* gene promoter reduced its activity, and pointed out that internal deletion of a sequence containing histone-specific motifs also reduced the promoter activity. Unlike Atanassova, Zheng and Murai [32] reported that any deletion from the 4.6 kb-long *glutelin* gene promoter region reduced the GUS activity in homologous rice but no distinct element was evident within the region by deletion analysis. So, these cases show that the deletion of promoter region associating with the change of transcriptional level depends on whether the deletion region contains distinct regulatory motifs and conceivable factors binding to the motif.

To further analyze the deletion and insert region necessary for transcriptional activation or suppression, we used a transient transactivation assay to identify candidate transcription factors (TFs) that were able to interact specifically with the different segment. After the search progress of various database, a data set of predicted transcription factors (Table S1) and putative binding site (Figure 1) was found in *PpDXS1* promoter. Interestingly, the deficiency segment of A16 promoter region contained a G-box site which was predicted to bind with the relevant G-box binding factors (GBFs). Generally, GBFs can be divided into three sub-groups involved in the two TFs superfamily, basic region-leucine zipper (bZIP) and basic helix-loop-helix (bHLH) types [33]. Long hypocothyl 5 (HY5) protein and phytochrome interacting factors (PIFs) are typical representatives of the two sub-groups of GBFs respectively [34]. Besides, proline-rich domain of GBF-1 protein (bZIP type) contribute to the function of activating transcription in Arabidopsis.

Considering the result of promoter-TFs prediction analysis, we verified whether candidate TFs (GBF1, CPRF1 and bHLH112 protein) bind to DXSpro_WT (containing the G-box site) through the luciferase protoplast assay. The resulting transactivation level showed there are little or no enhancements of the DXSpro_WT with *35S:bHLH112* and *35S:CPRF1*, in contrast with the observed enhancement when *35S:GBF1* was co-expressed in the *Poa pratensis* protoplast. We have demonstrated that the presence of GBF1 probably changes the transcription level, and we predicted that a G-box binding site is only located in the segment 3 of DXSpro_WT. After the yeast one-hybrid experiment, we also see the confirmed result that GBF1 only bind to the segment 3 of DXSpro_WT region (containing G-box site). Interestingly, at the same time, it has been recently reported that PIFs regulated the *DXS1* gene in *Arabidopsis*, probably through the PBE box (CACATG), whereas HY5 induces the expression of this gene through direct binding to the G-box containing core GCE element (ACGT) [34]. Importantly, we also provided evidence that overexpressing *GBF1* in transgenic *Poa pratensis* plant elevate the transcript level of *PpDXS1* gene. Hence together, our data support the assertion that deletion of the predicted G-box in the distal promoter region may account for the reduced transactivation levels and GBF1 are the strongest candidates to bind with G-box site of *DXS1* promoter in *Poa pratensis*.

Overall, our data suggest that a rearrangement in the upstream regulatory promoter region of the *DXS1* gene has significant impacts for the level of transcription in *Poa pratensis*, particularly due to the binding of GBF1 and deletion containing the G-box motif in the promoter region. While the significant differences of *DXS1* promoter and transcription regulation of GBF1 plays an important role in the multiple downstream pathways, such as chlorophyll and hormone synthesis, external condition and post-translational regulatory factors also control the plant growth and development [35].

## 4. Materials and Methods

### 4.1. Plant Materials and Growth Conditions

Kentucky bluegrass (*Poa pratensis* L.) cultivar ‘Baron’ was used as wild type (WT). A dwarf mutant selection (A16) was obtained from F3 dwarf plants derived from ‘Baron’ seeds exposed to space on the No. 20 Recoverable Science & Technology Satellite of China that flew in space for 18 days in 2004. Parameters of the satellite in orbit included the angle of 57–70°, perigee of 175–200 km, apogee of 300–400 km, orbit period of 90 min, and the microgravity level of 10^−3~−5^ *g*. During the 18 days of flight, the average accumulated dose in the cabin (excluding the ground control) was 2.656 mGy, and the average daily dose was 0.147 mGy/d. WT and A16 plants were grown in soil-sand-perlite (1:1:1, *v*/*v*) in plastic pots (16.5 cm height with 16.5 cm diameter) at 25 °C with 14/10-h day/night photoperiod.

### 4.2. Isolation and Analysis of PpDXS1 Promoter Region

Genomic DNA was extracted from WT and A16 leaves using a modified hexadecyl trimethyl ammonium bromide (CTAB) method. To clone the promoter region of *PpDXS1*, three high-efficiency thermal asymmetric interlaced polymerase chain reactions (hiTail-PCR) [36] were performed using degenerate primers and specific nested-primers (Table 1) designed against the *PpDXS1* coding sequence (MG257788). Amplified fragments produced by the three nested PCRs were cloned into pEASY-T1 cloning vector and then sequenced, named as T_DXSpro_WT and T_DXSpro_A16. Complete sequences (shown in Table S1) that extended upstream were generated containing 1649 bp and 1864 bp from the ATG of the *PpDXS1* coding sequence and used for further analysis. After the comparative analysis of the full-length sequences obtained, it was found that there was a rearrangement in the promoter region. On that basis the two sequences were divided into two common (Segment 1 and 2) and two unique segments (Segment 3 and 4).

The 5′ upstream sequences were analyzed for putative cis-acting regulatory elements using the PlantCARE database, and predicted for candidate transcription factor binding to promoter region by searching the TRANSFAC (Match public v1.0, www.gene-regulation.com) and JASPAR 2016 [37] databases. All candidate factors were applied filtering criteria (TRANSFAC: core match > 85% & matrix match > 85%, JASPAR: score > 10) and the above information were shown in Table S2.

### 4.3. Dual Luciferase Transient Protoplast Assay Using Poa pratensis Protoplast

To establish the transient expression system for promoter and transactive activity, we chose cultured young seedlings of KB for protoplast isolation. Protoplast were isolated as described by Junker et al. with some modification [38]. Briefly, 0.1 g 16-day-old young seedlings cultured at 25 °C on Murashige & Skoog (MS) medium with a 16 h light (~150 μmol·m^−2^·s^−1^)/8 h dark cycle, were used for protoplast separation. Leaf tissues cut into 1mm strips were immersed in enzymatic hydrolysate (2% cellulase, 1% pectinase, 1% macerozyme R-10 and 0.6 M mannitol). After digestion for 7 h in the dark at a speed of 40~80 r/min, W5 solution [39] of equal volume was slowly added to the mix, and then the liquid containing protoplast filtered through 200-mesh nylon was collected into a 50 mL-tube. The collected filtrate was centrifuged at 900 rpm for 10 min and supernatant was discarded. Then the purified protoplast was obtained after washing it twice with W5 solution, and resuspended with Mineral modified Glutamate (MMG) solution [39]. The quantity and quality of protoplast were detected under a microscope.

To determine the activity of the promoter segment, individual segments and combined fragments were generated from the T_DXSpro_WT and T_DXSpro_A16 vector, including Segment_1 (608 bp), Segment_1+2 (1149 bp), Segment_1+4 (1323 bp), Segment_1+2+3 (1649 bp), and Segment_1+4+2 (1864 bp). These fragments were subcloned into the pGreen_0800_*luc* transient expressed vector incorporated *XhoI* and *HindIII* restriction sites (primers were shown in Table S3). Polyethylene glycol (PEG)-mediated transfections were carried out as described by Zhang et al. [39]. Finally, the protoplasts were transferred into multi-well plates and cultured under dark at 25 °C for 24 h. The protoplasts after induced culture were tested for the activity of firefly luciferase (Fluc) reporter gene in TECAN infinite M200 PRO (Tecan, Mannedorf, Switzerland) according to the instructions of double-luciferase reporter assay kit (Transgen Biotechnology, Beijing, China). Renilla luciferase (Rluc) was co-expressed in all experiments, serving as an internal control, and Fluc activity was normalized to Rluc activity. The luciferase experiments were repeated independently at least three times, taken the transferred pGreen_0800_*luc* vector as positive control and the untransformed protoplast as negative control.

The above method was further applied for investigation promoter-protein interactions by luciferase assays. The recombinant plasmids of Segment_1+2+3_Fluc (also namely as DXSpro_WT_Fluc) were used as the reporter gene in this study. Besides, *G-box binding factor 1* (*PpGBF1*, MK327144), *common plant regulatory factor 1* (*PpCPRF1*, MK327145) and *basic helix-loop-helix factor 112* (*PpbHLH112*, MK327146) genes were cloned from KB cDNA without the stop codon and inserted into pGreen_62_SK (primers with restriction sites are shown in Table S3). For co-expression assays of promoter and transcription factors, the total plasmid DNA was between 10 μg and 15 μg. Protoplast transfection and Fluc detection analysis were the same as the above promoter activity protocol.

### 4.4. Yeast One-Hybrid Assay

To further identify the promoter segment of protein-DNA interaction, based on above work, *PpDXS1* promoter segments were inserted individually into the pAbAi vector with primers (Table S3) and formed plasmids of pDXSpro_1/2/3/4_AbAi which were transformed into Y1HGold strain for screening the optimal resistance concentrations of AbA. The transcription factor genes (*GBF1*, *CPRF1*, and *bHLH112*) with restriction sites (Table S3) were ligated into the pGADT7 vector to construct the recombinant plasmid of pGADT7-TFs. Then the pGADT7-TFs were individually transformed into Y1H (pDXSpro_1/2/3/4_AbAi) for examing the interaction between TF protein and individual promoter segments of *PpDXS1* on media lacking Leu (SD/-Leu) supplemented with optional AbA of 500 ng/mL, 350 ng/mL, 250 ng/mL and 200 ng/mL, respectively. The experiment procedure was carried out in accordance with the product manual (Takara Biomedical Technology Co., Ltd., Beijing, China).

### 4.5. Transformation of Poa pratensis and GUS Histochemical Assay

The full-length promoter region from WT and A16 were individually cloned into pCAMBIA1301 binary vector incorporated *Hind*III and *Nco*I restriction sites (Table S3), named as DXSpro-WT:*GUS* and DXSpro-A16:*GUS*. Besides, based on above study, the *GBF1* gene was also cloned into pTCK303 vector incorporated *Bam*HI and *Sac*I restriction sites (Table S3), named as 303_OE_*GBF1*. The recombination vectors were used to generate transgenic line through particle bombardment-mediated transformation into KB embryogenic calli [40]. Transgenic lines of the transformed CaMV 35S:*GUS* vector was the positive control and the wild-type KB was the negative control. All the transformed callus was transferred to MS selection medium supplemented with 100 mg/L hygromycin under dark conditions. After two selection rounds, hygromycin resistance callus was transferred to regeneration medium supplemented with 50 mg/L hygromycin and incubated under a 16/8-h (light/dark) photoperiod. Fully recovered plantlets were transferred to containers for further root development and, finally, green plants were transferred to soil for growth. Transgenes were identified in T0 plants by PCR with three pair primers for the gene specific primer plus vector primer, two universal vector primers and 35S promoter regions (Table S3).

To further identify the positive of transgenic seedling and also observe the difference in promoter activity, 21-day-old shoots were stained using the GUS histochemical assay. Plant tissues were visualized using a stereoscopic microscope (LEICA M205FA, Buffalo Grove, IL, USA)

### 4.6. Expression Analysis

Total RNA from leaves was obtained TRIzol reagent kit (Invitrogen, Carlsbad, CA, USA) using 100~500 mg tissue homogenized in liquid nitrogen according to the protocol. Its quantity and purity were assessed using the NanoDrop 2000 (Thermo, Waltham, MA, USA) and reverse transcriptional reaction was carried out with 0.5 µg total RNA using PrimeScript™ RT reagent Kit (Perfect Real Time) (Takara Biomedical Technology Co., Ltd., Beijing, China) according to the supplier’s instruction.

Real-time PCR was carried out using a SYBR Green assay (Takara, Dalian, China) on a Bio-Rad CFX96 System (BIO-RAD, Hercules, CA, USA). Each 10 µL assay contained 5 µL SYBR Premix Ex Taq, 1 µL cDNA and 100 nM of each primer (Table S3). Analysis were done with three independent experiments and technical duplicates were included in each case (*n* = 3). The relative mRNA abundance was calculated by using the comparative *C*_t_ method (2^−ΔΔ*C*t^) and normalization to actin gene. Besides, *pgk1* gene is known as the single copy gene in Poaceae family, so it was used to determine the copy number of transgenic gene (*GUS* and *GBF1*) by the formula of *GUS* or *GBF1* gene *C*_t_ value/*pgk1* gene *C*_t_.

### 4.7. Statistical Analysis

Statistical analysis was performed in SPSS Statistics 19 (Chicago, IL, USA) using analysis of variance (ANOVA) and values *p* < 0.05 were deemed statistically significant.

## Figures and Tables

**Table 1:** Specific nested-primers for PpDXS1 promoter amplication.

Primer Name	Sequence (5′-3′)
SP1	CCGTCGGTCTGCCGCATCGTC
SP2	ACGATGGACTCCAGTCGCTTGTCCTGAGGGGTGTTG
SP3	TGAGGGACAGGTTCTTCATGTGGA
SP4	CCTCTCCGTCAGCGACGCAGAAA
SP5	ACGATGGACTCCAGATCTGGGGAGTGGAGCGGGA
SP6	CGTGTGGCTGGTATTCGTTCG
SP7	TCGGGACAGGGAGAGCGAAATGG
SP8	ACGATGGACTCCAGAAAAGAGCAAAGGGGAACCG
SP9-WT	GAACAAAGTGGAATGTCGGCG
SP9-A16	TGTGGAGGAGGTGGTGGTGCC

## Supplementary Materials

Supplementary materials can be found at https://www.mdpi.com/1422-0067/20/6/1398/s1. **Table S1.** The complete sequences of *PpDXS1* promoter isolated from WT and A16. **Table S2.** Analysis of cis-acting regulatory element in *PpDXS1* promoter and putative transcription factor binding to *PpDXS1* promoter predicted by PlantCARE, TRANSFAC and JASPAR2016 online database. **Table S3.** List of various primers and their uses. **Table S4.** Detected copy results of *GUS* gene of transgenic lines. **Figure S1.** GUS histochemical staining of transgenic lines, including DXSpro_WT, DXSpro_A16, pCAMBIA1301 control. **Figure S2.** The result of interaction between other segment of *PpDXS1* promoter region and transcription factor in yeast one hybrid assays.
